# Characteristics of acid‐sensing ion channel currents in male rat muscle dorsal root ganglion neurons following ischemia/reperfusion

**DOI:** 10.14814/phy2.15654

**Published:** 2023-03-26

**Authors:** Qin Li, Lu Qin, Jianhua Li

**Affiliations:** ^1^ Heart and Vascular Institute The Pennsylvania State University College of Medicine Hershey Pennsylvania USA

**Keywords:** acid‐sensing ion channels, dorsal root ganglion, ischemia–reperfusion, peripheral artery disease

## Abstract

Peripheral artery diseases (PAD) increases muscle afferent nerve‐activated reflex sympathetic nervous and blood pressure responses during exercise (termed as exercise pressor reflex). However, the precise signaling pathways leading to the exaggerated autonomic responses in PAD are undetermined. Considering that limb ischemia/reperfusion (I/R) is a feature of PAD, we determined the characteristics of acid‐sensing ion channel (ASIC) currents in muscle dorsal root ganglion (DRG) neurons under the conditions of hindlimb I/R and ischemia of PAD. In particular, we examined ASIC currents in two distinct subpopulations, isolectin B_4_‐positive, and B_4_‐negative (IB4+ and IB4−) muscle DRG neurons, linking to glial cell line‐derived neurotrophic factor and nerve growth factor. In results, ASIC1a‐ and ASIC3‐like currents were observed in IB4− muscle DRG neurons with a greater percentage of ASIC3‐like currents. Hindimb I/R and ischemia did not alter the distribution of ASIC1a and ASIC3 currents with activation of pH 6.7 in IB4+ and IB4− muscle DRG neurons; however, I/R altered the distribution of ASIC3 currents in IB4+ muscle DRG neurons with pH 5.5, but not in IB4− neurons. In addition, I/R and ischemia amplified the density of ASIC3‐like currents in IB4− muscle DRG neurons. Our results suggest that a selective subpopulation of muscle afferent nerves should be taken into consideration when ASIC signaling pathways are studied to determine the exercise pressor reflex in PAD.

## Graphical abstract

Ischemia and ischemia–reperfusion affect acid‐sensing ion channels in muscle sensory neurons. The results suggest that acid‐sensing ion channel type 3 plays a major role in regulating the exercise pressor reflex at the cellular level in peripheral artery disease.

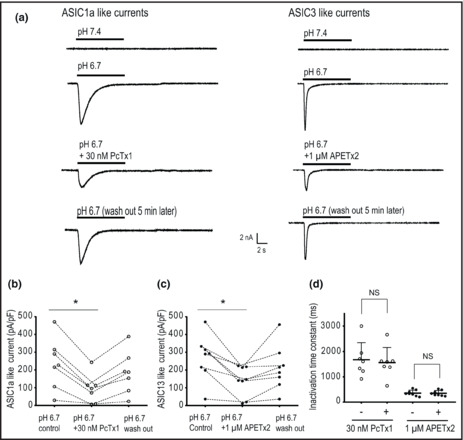

## 1 INTRODUCTION

Peripheral artery disease (PAD) is a common and disabling disease affecting over 200 million worldwide (Criqui & Aboyans, 2015; Fowkes et al., 2017). In addition to the walking limit due to “intermittent claudication” (Hart et al., 2018), PAD patients are at a high risk of myocardial infarctions, cerebral vascular accidents, and all‐cause mortality as blood pressure (BP) response is increased during exercise (Anand et al., 2018; Bauersachs et al., 2019; Ouriel, 2001). Note that an exaggerated BP driven by the sympathetic nervous activity (SNA) contributes to poor clinical outcomes (Piepoli et al., 1999; Ponikowski et al., 2001), associated with decreased survival in PAD patients (de et al., 2008).

The two major neural regulatory mechanisms are thought to contribute to sympathetic engagement of BP response during exercise, namely “Exercise Pressor Reflex” and “Central Command” (Coote et al., 1971; Mitchell et al., 1983; Waldrop et al., 1996). Despite other neural mechanisms involved in BP regulation during exercise, such as arterial baroreflex, the exercise pressor reflex is a major determinant of why BP is exaggerated during exercise in PAD patients (Baccelli et al., 1999; Bakke et al., 2007; Ritti‐Dias et al., 2011).

The exercise pressor reflex involves metabolic stimulation (i.e., “metaboreceptor” stimulation) and to mechanical deformation of the muscle afferents receptive field (i.e., “mechanoreceptor stimulation”) (Kaufman, 1996). When the receptors in the free endings in muscle interstitium are stimulated, unmyelinated group IV (predominantly metabosensitive) and myelinated group III (predominantly mechanically sensitive) afferents arising from contracting skeletal muscle are engaged, cardiovascular nuclei in the brainstem are activated and the SNA increases and BP rises (Kaufman, 1996; Mitchell et al., 1983). Using animal models of PAD, a number of signal pathways and receptors are suggested to play a role in regulating the exaggerated exercise pressor reflex, that is, increases in products of oxidative stress and proinflammatory cytokines, and dysfunction of acid‐sensing ion channels (ASICs), purinergic P2Xs, and bradykinin receptors (Liu et al., 2010; Stone & Kaufman, 2015; Teixeira & Vianna, 2022; Xing et al., 2012). Of note, some of mechanisms are found in human studies (Campos et al., 2019; Muller et al., 2012). Nonetheless, the underlying signaling pathways at cellular and molecular levels by which the exercise pressor reflex is exaggerated in PAD are necessary to be studied to shed lights on clinical human studies.

Specifically, contracting skeletal muscles during exercise induce an accumulation of numerous metabolites in the interstitial space of the activated muscles, such as lactic acid, along with decreased pH levels, which stimulate ASICs on the free endings of group III and IV muscle afferents residing in the interstitium (Kaufman & Hayes, 2002; Sinoway & Li, 2005). Lactate, acidic phosphate, and H^+^ are the endogenous modulators to ASICs playing a role in the exercise pressor reflex (Immke & McCleskey, 2001; Osmakov et al., 2014) (Campos et al., 2019; Hayes et al., 2007, 2008; Liu et al., 2010; Stone et al., 2015; Stone & Kaufman, 2015; Tsuchimochi et al., 2011). In animal studies, ASIC1a and ASIC3 are shown to participate in the reflex and ASIC3 is identified to contribute to the exaggerated reflex in a rat model of PAD (Farrag et al., 2017; Kim et al., 2020; Xing et al., 2012).

Rats with femoral artery ligation are used to study the responses of SNA and BP to exercise (Kim et al., 2020; Li et al., 2020, 2021; Liu et al., 2010; Stone et al., 2015; Tsuchimochi et al., 2011; Xing et al., 2012) (Waters et al., 2004). This rat model replicates a condition of insufficient blood flow to the contracting leg muscles during exercise in PAD patients. Besides the ischemia phase during exercise in PAD, a recovery of the blood supply to the limb muscles during resting results in ischemia–reperfusion (I/R) injury (Vun et al., 2016). Indeed, femoral artery ischemia per se in this rat model may not totally reflect pathophysiology of the exercising muscles in PAD patients. Moreover, the cellular and molecular mechanisms leading to ischemia and I/R injury are distinct in PAD (Hamburg & Creager, 2017; Simon et al., 2018), which are likely to differently affect the exercise pressor reflex in animals with hindlimb I/R and femoral artery occlusion (FAO). Thus, it is significant to study the exercise pressor reflex under both conditions in PAD. The purposes of the present study were (1) to examine the effect of the hindlimb I/R and FAO on ASIC currents (i.e., ASIC1a and ASIC3) in rat muscle dorsal root ganglion (DRG) neurons and (2) to further characterize the current activities in two different populations of muscle afferent nerves, namely isolectin B_4_‐positive and isolectin B_4_‐negative (IB4+ and IB4−) muscle DRG neurons. IB4+ neurons express receptors for glial cell line‐derived neurotrophic factor (GDNF), whereas IB4− DRG neurons are depending on nerve growth factor (NGF) for survival (Averill et al., 1995; Bennett et al., 1998; Michael et al., 1997; Molliver et al., 1997). We hypothesized that PAD upregulates ASIC signaling pathways in a subpopulation of NGF‐involved muscle afferent nerves, being potentially responsible for the exaggerated exercise pressor reflex.

## 2 MATERIALS AND METHODS

### 2.1 Ethics statement

All experimental procedures were approved by the Institutional Animal Care and Use Committee of Penn State College of Medicine (Protocol#: PRAMS201147671) and were conducted in accordance with the National Institutes of Health Guide for the Care and Use of Laboratory Animals. Male Sprague–Dawley rats (4–6 weeks old) were housed in accredited temperature and ventilation‐controlled facilities with a 12:12‐h light–dark cycle and ad libitum access to standard rat chow and water.

### 2.2 Hindlimb ischemia and reperfusion

As described previously (Li et al., 2020, 2021), in order to make femoral artery ischemia, the rats were anesthetized by inhalation of an isoflurane–oxygen mixture (2%–5% isoflurane in 100% oxygen). The femoral artery on one limb was surgically exposed, dissected, and ligated ~3 mm distal to the inguinal ligament as FAO group. The incision and skin were closed with surgical staples. For hindlimb I/R group, the same ligation procedures were performed, and then, the incision and skin were closed with surgical staples. The rats were kept in normal air of the surgery room without anesthesia for 6 h and then by inhalation of an isoflurane–oxygen mixture the ligation of femora artery was reopened to recover the blood flow into the femoral artery and the surgical incision and skin were closed. The control rats were dealt with the same procedures except that a suture was placed below the femoral artery, but the artery was not tied. After the surgery, the FAO rats were returned to the cage for 24‐h regular housing and I/R rats for 18 h before the electrophysiological experiments.

Note that buprenorphine hydrochloride (0.05 mg/kg, subcutaneously) was administered prior to the surgery for postoperative pain relief. Following the surgery, the animals were kept in the surgery room for 2–3 h for observation and then returned to the animal facility.

### 2.3 Labeling of hindlimb muscle afferent DRG neurons

As described previously (Li et al., 2020, 2021), 2 days before FAO and I/R models were constructed, an incision in the calf area of one limb was made and the gastrocnemius muscle was exposed following rats were anesthetized. The lipophilic dye 1, 1′‐dioctadecyl‐3, 3, 3′, 3′‐tetramethylindocarbocyanine perchlorate (DiI, 60 mg/mL) was injected into the white portion of the gastrocnemius muscle. A total volume of 1 μL DiI tracer was administered at different locations, with the needle left in the muscle for 1 min to prevent the tracer leakage. The same procedure was made in the contralateral limb. After that, the rats were returned to their cages for the fluorescent DiI retrograde transported to DRGs to label muscle DRG neurons.

### 2.4 Culture of DRG neurons

To euthanatize rats, they were anesthetized with over 5% isoflurane 24 and 18 h after the surgery for FAO group and for I/R group, respectively, and then were decapitated. After this, L4–L6 DRGs in both sides were removed and dissected and then immediately transferred into ice‐cold Hank's balanced salt solution (HBSS). After being freed from the connective tissues, the ganglia were enzymatically digested and dissociated in Earle's balanced salt solution (Sigma‐Aldrich) containing collagenase Type D (0.6 mg/mL; Roche), trypsin (0.30 mg/mL; Worthington) and DNase (0.1 mg/mL; Thermo), followed by shaking for 45 min at 34°C. The dissociated neurons were seeded on 10% poly‐L‐lysine‐coated coverslips (Dia^#^ 8 mm) in a 35‐mm culture dish containing 2 mL DMEM medium (Thermo) supplemented with 10% FBS, 1% glutamine, and 1% penicillin–streptomycin. Then, the neurons were cultured at 37°C with 5% CO_2_, 95% air in a cell culture incubator (VWR).

### 2.5 Electrophysiology

All patch clamp recording was performed on the DRG neurons within 6 h after their being dissociated. Immediately before recording, neurons were incubated with IB_4_‐Alexa Fluor 488 (3 μg/mL in external solution, Invitrogen) for 5 min and then rinsed with external solution for another 5 min. Two distinct subpopulations of neurons namely IB4+ and IB4− muscle DRG neurons were identified under a microscopy. that is, DRG neurons were first visualized using differential interference contrast (DIC; x20‐40) optics, and then, an IB4+ neuron was visualized (as green color) using a combination of fluorescence illumination and DIC optics on a Nikon TE2000 inverted microscope. Meanwhile, those DRG neurons were also identified as Dil‐positive (red color) under an inverted microscope with a fluorescent filter, and images were displayed on a video monitor. Then, IB4+ muscle DRG neurons were identified and IB4+ and IB4− muscle DRG neurons were randomly obtained from 20 fields in five coverslips under the microscopy to obtain enough numbers of neurons from each group for statistical analysis.

ASIC currents of muscle DRG neurons (cell diameters ≤35 μM) were recorded in the whole‐cell configuration using a MultiClamp 700B amplifier supplied with Digitizer 1440A (Axon Inc). After pH 7.6, pH 6.7, and pH 5.5 were applied (not sequentially), each recording was obtained from one individual muscle DRG neuron of control, I/R, and FAO groups, respectively. Signals were acquired with pClamp10.1 and analyzed with pClampfit 10.7 software. All experiments were performed at room temperature of 20–22°C. Neurons were considered proton‐sensitive for analysis if acid solution elicited an inward current of >50 pA in peak amplitude.

Modified from Liu and Oliver (Liu et al., 2004; Poirot et al., 2006), the extracellular solutions contained 140 mM NaCl, 5 mM KCl, 2 mM CaCl_2_, 1 mM MgCl_2_, 10 mM Mes (2‐(N‐morpholino) ethanesulfonic acid), 10 mM glucose and pH was adjusted to 5.5 and 6.7 with 1 M NaOH, respectively. Holding at −70 mV, after the seals (2 ~ 8 GΩ) were obtained with 3 ~ 5 MΩ resistance of glass electrodes filled with internal solution (in mM): 140 KCl, 2 MgCl2, 5 EGTA, 10 HEPES, 0.3 NaGTP and 2 MgATP, pH adjusted to 7.3 using 1 M KOH. The whole‐cell configuration was applied, and series resistance was 70%–90% compensated to under 10 MΩ if necessary. In voltage‐clamp mode, one gap‐free protocol of 30 s was used to record ASIC currents. All the chemicals stored in the stock solutions were diluted in extracellular solution immediately before being used and individually held in a series of independent syringes of the pressurized VC3‐8MP perfusion system with an ID#200 μm outlet tip (ALA Inc.). The distance from the outlet tip mouth to the neuron examined was within 100 μm. The DRG neurons in the recording chamber were continuously bathed in extracellular solution of pH 7.4.

### 2.6 Statistical analysis

All data in this study were presented as mean ± SD (standard deviation). Paired *t*‐test was used for ASIC1a‐like currents and ASIC3‐like currents when antagonists applied. A two‐way or three‐way chi‐squared test (χ^2^ test) was used to analyze the distribution of the neurons with ASIC currents, ASIC1a‐like, and ASIC3‐like currents. One‐way or two‐way ANOVA was applied to analyze the current density of ASIC1a‐like or ASIC3‐like currents, and post hoc analysis with Tukey's tests was applied to compare the difference between specific groups. All statistical analyses were performed using SPSS v26, and the significant differences were considered at *p* < 0.05.

## 3 RESULTS

### 3.1 Determination of ASIC1a and ASIC3 currents in muscle DRG neurons

The pH range required to activate ASIC3 is approximately 6.5–7.0 (Deval et al., 2008, 2011), which is close to what is observed in exercising muscle and/or moderately ischemic tissues (MacLean et al., 1998, 2000; Rotto et al., 1989; Yagi et al., 2006). A lower pH is likely to stimulate more ASIC subtypes seen in the process of pathophysiological responses such as ischemic insult and/or I/R injury. Currently, it has been accepted that ASIC1a and ASIC3 exist in the sensory nerves and are engaged in modifying sensation and nociception observed in various experimental models. Thus, in this study we selected pH 6.7 and pH 5.5 to stimulate ASICs and examined the effects of IR injury and ischemia of PAD on ASIC currents.

**FIGURE 1:**
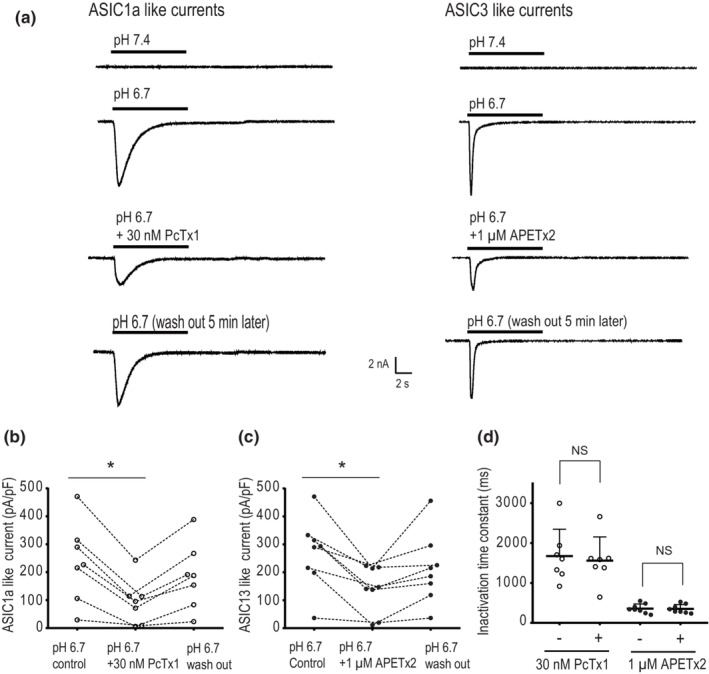
ASIC currents in rat muscle DRG neurons. (a) The representative traces of ASIC1a‐like currents (left) and ASIC3‐like currents (right) in rat muscle DRG neurons at pH 6.7 before and after 30 nM PcTx1 (antagonist to ASIC1a channels), and 1 μM APETx2 (antagonist to ASIC3 channels) applied for 10 s. PcTx1 and APETx2 were pre‐applied for 1 min, followed by application of pH 6.7 for 10 s, respectively. (b) The aligned plot of the current density of ASIC1a‐like currents before and after 30 nM PcTx1 applied. *p < 0.05, comparing between pH 6.7 and pH 6.7 plus antagonist with a paired *t‐test* analysis. (c): The aligned plot of the current density of ASIC3‐like currents before and after 1 μM APETx2 applied. *p < 0.05, comparing between pH 6.7 and pH 6.7 plus antagonist with a paired *t*‐test analysis. (d) The inactivation time constants of ASIC1a‐like currents and ASIC3‐like currents at pH 6.7 before and after application of 30 nM PcTx1 and 1 μM APETx2, respectively. NS indicates no significant difference (analyzed with a paired *t*‐test) observed in inactivation time constants of ASIC1a and ASIC3 currents before and after the application of their respective antagonists.

When pH 6.7 and pH 5.5 solutions were applied for 10 seconds, two distinct types of inward currents were observed in rat muscle DRG neurons, termed as ASIC1a‐like and ASIC3‐like currents in this study. They were distinguished by their inactivation time constant (*τ*_inact_). When fitting with standard exponential function, with pH 6.7 ASIC1a‐like currents had a slower *τ*_inact_ of 1673.54 ± 672.62 ms (*n* = 7) and ASIC3‐like current had a faster *τ*_inact_ of 369.46 ± 114.87 ms (*n* = 8). In addition, the amplitude of ASIC1a was decreased by 65.94 ± 14.24% with 30 nM PcTx1 (antagonist to ASIC1a channels; (Figure 1a left panel & Figure 1b)). The amplitude of ASIC3 was attenuated by 52.5 ± 20.87% when 1 μM APETx2 (antagonist to ASIC3 channels) was applied (Figure 1a right panel & Figure 1c). There was no difference in *τ*_inact_ (*p* = 0.902) before and after application of PcTx1 and *τ*_inact_ (*p* = .879) before and after application of APETx2 (Figure 1d). These results are consistent with the previous reports showing that functional ASIC1a and ASIC3 channels were expressed and activated in rat muscle DRG neurons (Farrag et al., 2017; Xing et al., 2012). However, it should be noted that a less % attenuation of PcTx1 and APETx2 on ASIC currents observed in the current study was likely due to the activation of heteromeric ASICs after application pH 6.7 and pH 5.5 solutions.

### 3.2 Effect of hindlimb I/R and ischemia on distribution of IB4+ and IB4− muscle DRG neurons

**FIGURE 2:**
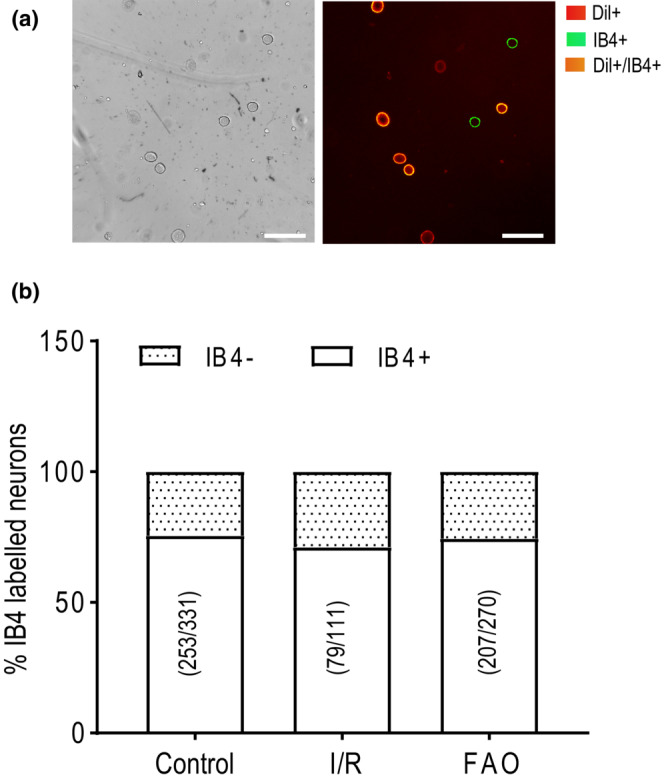
Effect of hindlimb I/R and ischemia on the distribution of IB4+ and IB4− muscle DRG neurons. (a) The representatives of IB4+ muscle DRG neurons (orange), IB4− muscle DRG neurons (red), and IB4+ DRG neurons with no DiI label (green). Scale bar: 50 μm. (b) The histogram shows the proportion of IB4+ and IB4− muscle DRG neurons in the control, I/R, and FAO groups. IB4+ and IB4− muscle DRG neurons were randomly numbered from 20 version fields in five coverslips. The number of IB4+, IB4− neurons, and total counted neurons was listed in histogram. No significant difference among the groups when analyzed with chi‐squared test.

As shown in Figure 2a, muscle DRG neurons were divided into IB4− neurons (labeled red) and IB4+ neurons (labeled orange), identified with red and green fluorescent filters under a microscopy. 75.56% of muscle DRG neurons (235 out of 311 counted) in the control rats was IB4+, and merely 24.44% of neurons was IB4−. As shown in Figure 2b, when compared to the control rats, I/R and FAO had no effect on the distribution of IB4+ muscle DRG neurons and IB4− muscle DRG neurons (χ^2^ = 0.828, *p* = 0.660).

### 3.3 Effect of hindlimb IR and ischemia on distribution of ASIC currents

**FIGURE 3:**
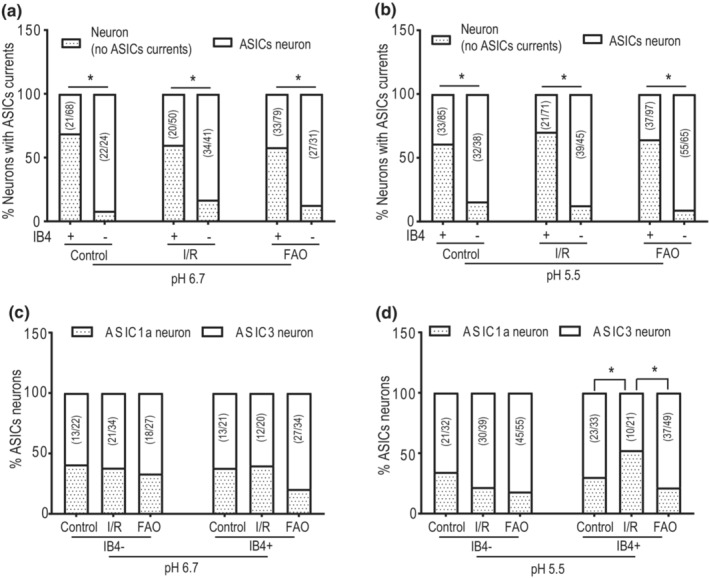
Effect of hindlimb I/R and ischemia on the distribution of ASIC1a and ASIC3 currents in rat muscle DRG neurons. (a, b) The distribution of ASIC currents in IB4− and IB4+ rat muscle DRG neurons at pH 6.7 and pH 5.5 in the control, I/R, and ischemia groups. The number of ASIC neurons and total recorded neurons were shown in histograms. *p < 0.001 between IB4− neurons and IB4+ neurons in each group when analyzed with chi‐squared test. (c, d) The distribution of ASIC1a‐ and ASIC3‐like currents in IB4− and IB4+ muscle DRG neurons in the control, I/R, and FAO groups. *p < 0.05 between the two groups indicated, using analysis of chi‐squared test.

To identify the characteristics of ASIC currents in rat muscle DRG neurons, we further examined ASIC1a and ASIC3 currents in rat muscle DRG neurons of IB4− group and IB4+ group. Overall, ASIC currents were largely seen in IB4− muscle DRG neurons with activation of pH 6.7 and pH 5.5 (Figure 3a,b). In the control rats, application of pH 6.7 induced 91.67% of IB4− muscle DRG neurons (22/24) to generate ASIC currents (termed as ASIC neuron in this study), but merely 30.88% in IB4+ neurons (21/68; *p* < 0.001 between IB4− neurons and IB4+ neurons). In addition, as shown in Figure 3a, at pH 6.7, % ASIC neurons in I/R group was 82.93% in the recorded IB4− neurons (34/41), but 40.00% in IB4+ neurons (20/50; *p* < 0.001 between IB4− neurons and IB4+ neurons). In FAO group, % ASIC neurons were 87.10% in the recorded IB4− neurons (27/31) and 41.77% in IB4+ neurons (33/79; *p* < 0.001 between IB4− neurons and IB4+ neurons). No difference was found in % ASIC neurons among the control, I/R, and ischemia groups either in the recorded IB4− neurons (χ^2^ = 1.004, *p* = 0.605) or IB4+ neurons (χ^2^ = 2.017, *p* = 0.365). At pH 5.5, the similar distribution pattern of % ASIC neurons was observed, and this was not affected by hindlimb I/R and FAO (Figure 3b). The data suggested that ASIC currents were mainly distributed in IB4− muscle DRG neurons, and either I/R or ischemia did not alter the distribution of ASIC currents in IB4− and IB4+ muscle DRG neurons.

Moreover, as shown in Figure 3c, in the control rats, at pH 6.7, % ASIC3 neurons was 59.10% (13/22) in IB4− muscle DRG neurons, whereas % ASIC1a neurons was merely 40.90%. The similar proportion pattern was also found in IB4+ muscle DRG neurons, that is, % ASIC3 neurons and % ASIC1a neurons were 61.90% and 38.10%, respectively. No difference was observed between IB4− ASIC neurons and IB4+ ASIC neurons (χ^2^ = 0.036, *p* = 0.850). Further analysis shows that, at pH 6.7, I/R and FAO had no effect on the distribution ASIC1a and ASIC3 in IB4− ASIC neurons (χ^2^ = 0.317, *p* = 0.854) and IB4+ ASIC neurons (χ^2^ = 2.989, *p* = 0.224), as compared to their respective controls. In addition, in IB4+ ASIC neurons, at pH 5.5, % ASIC3 neurons and % ASIC1a neurons were respectively 47.62% and 52.38% in I/R group, whereas 73.38% and 21.62% in FAO group (χ^2^ = 5.859, *p =* 0.015 between two groups; Figure 3d). Meanwhile, it was also found that there was the difference between the control and I/R group (χ^2^ = 4.107, *p* = 0.043 between two groups). However, no difference was found in IB4− ASIC neurons among the groups (χ^2^ = 2.941, *p* = 0.230). The data indicated that I/R and ischemia had no effect on the distribution ASIC3‐like currents and ASIC1a‐like currents in IB4− muscle DRG neurons with activation of pH 5.5. However, in IB4+ muscle DRG neurons, I/R increased the distribution of ASIC1a currents.

### 3.4 Effect of hindlimb I/R and ischemia on the amplitude of ASIC currents in rat muscle DRG neurons

**FIGURE 4:**
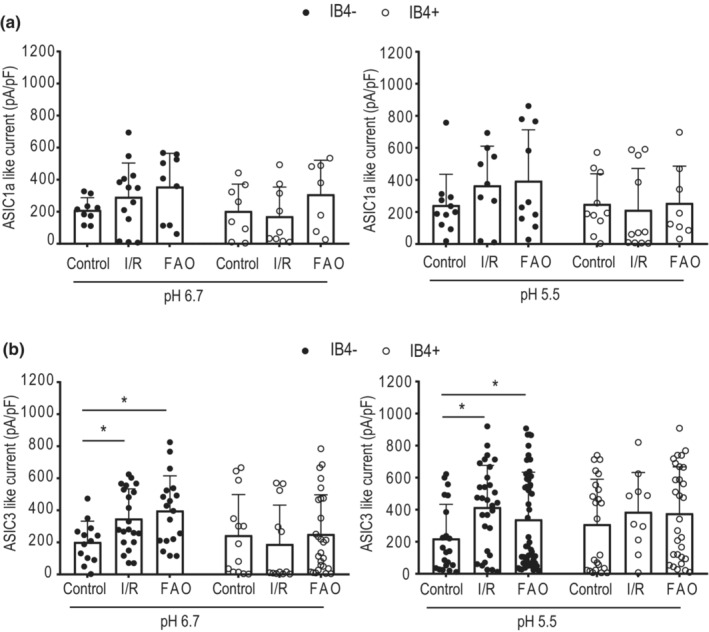
Effect of hindlimb I/R and ischemia on the activities of ASIC currents in rat muscle DRG neurons. (a) The histogram of the averaged current density of ASIC1a‐like currents in IB4− and IB4+ muscle neurons at pH 6.7 (left) and pH 5.5 (right) in the control, I/R, and FAO rats. When analyzed with ANOVA, results show no significant difference observed among the three groups either application of pH 6.7 or pH 5.5 solution. (b) The histogram of the averaged current density of ASIC3‐like currents in IB4− and IB4+ muscle neurons at pH 6.7 (left) and pH 5.5 (right) in the control, I/R, and ischemic rats. *p < 0.05 between the two groups when analyzed with ANOVA. There was no significant difference in current density of ASIC3‐like currents in IB4+ DRG neurons observed among the three groups either application of pH 6.7 or pH 5.5 solution. Note that the induced inward currents >50 pA when pH 6.7 or 5.5 applied were analyzed.

**FIGURE 5:**
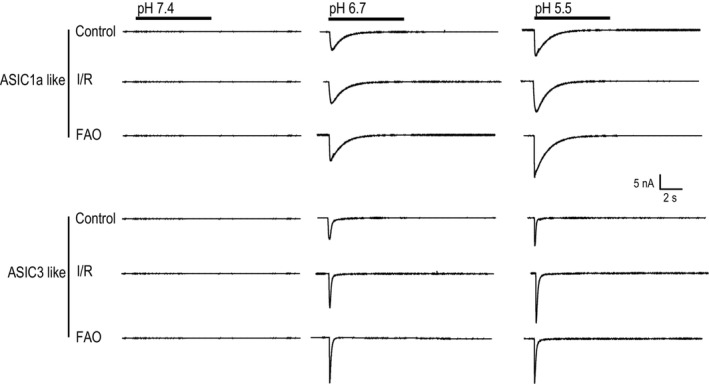
Representative traces of ASIC1a‐like currents and ASIC3‐like currents in IB4− muscle DRG neurons of the control, I/R, and FAO groups. Each trace was recorded from one individual muscle DRG neuron of control, I/R, and FAO.

As shown in Figure 4a (left panel), at pH 6.7, both I/R and FAO tended to amplify ASIC1a‐like current in IB4− muscle DRG neurons (control: 211.30 ± 75.89 pA/pF/*n* = 9, I/R: 293.49 ± 211.39 pA/pF/*n* = 13 and FAO: 357.12 ± 207.09 pA/pF/*n* = 9), as well as in IB4+ neurons (control: 205.35 ± 167.06 pA/pF/*n* = 8, I/R: 172.50 ± 181.89 pA/pF/*n* = 9, and FAO: 309.84 ± 211.81 pA/pF/*n* = 7). However, no significant difference in the density of ASIC1a currents was found among the groups (*F* = 1.396, *p* = 0.247). Similarly, no significant difference was observed among the control, I/R, and FAO groups (F = 0.943, *P* = 0.463) with pH 5.5 applied as shown in Figure 4a (right panel). The representative traces of ASIC1a current are illustrated in Figure 5 (top panel).

Figure 4b (left panel) shows that at pH 6.7, hindlimb I/R and FAO significantly amplified ASIC3‐like currents in rat muscle DRG neurons. The density of ASIC3‐like currents in IB4− muscle DRG neurons of I/R group was 349.64 ± 185.10 pA/pF (*n* = 21) (comparing to the control group of 203.26 ± 129.62 pA/pF/n = 13, *p* = 0.027), and 399.63 ± 216.04 pA/pF in FAO group (*n* = 18, *p* = 0.010 comparing to the control). However, no significant difference in the density of ASIC3‐like currents was found in IB4+ muscle DRG neurons among the groups (*p* = 0.765). On the contrary, at pH 5.5, both I/R and FAO also significantly increased ASIC3‐like current in IB4− muscle DRG neurons (*p* = 0.024) as shown in Figure 4b (right panel). The representative traces of ASIC3 current are illustrated in Figure 5 (bottom panel). The data indicated that both hindlimb I/R and FAO amplified ASIC3‐like currents in IB4− muscle DRG neurons.

## 4 DISCUSSION

In PAD rats, 24–72 h of femoral occlusion increases ASIC3 expression in L4‐6 DRGs accompanied with the increases in ASIC3 current activities in muscle DRG neurons (Farrag et al., 2017; Liu et al., 2010; Xing et al., 2012). ASIC1a also plays a role in the exercise pressor reflex in normal rats (Ducrocq et al., 2020a, 2020b). A study using ASIC3 KO rats confirms the role played by ASIC3 in the exaggerated reflex in PAD (Kim et al., 2020). Interestingly, a recent human study further suggests that ASICs are involved in muscle afferent mediated‐exercise pressor reflex (Campos et al., 2019). Thus, in the present study, it is important to determine the characteristics of ASIC currents in IB4+ and IB4− rat muscle DRG neurons, which represent two subgroups of DRG neurons having distinct electrophysiological characteristics (Averill et al., 1995; Bennett et al., 1998; Michael et al., 1997; Molliver et al., 1997) (Liu et al., 2004; Poirot et al., 2006; Stucky & Lewin, 1999). We also determined the effect of hindlimb I/R and ischemia seen in PAD on ASIC1a and ASIC3 currents. The main novel findings include that (1) ASIC currents are mostly present in IB4− muscle DRG neurons, and ASIC3‐like currents are prominent over ASIC1a‐like currents; (2) hindlimb I/R and FAO increase the activities of ASIC3 currents in IB4− muscle DRG neurons with activation of pH 6.7 and pH 5.5; and (3) hindlimb I/R alters the percentage distribution of ASIC1a currents in IB4+ muscle DRG neurons with pH 5.5, but not in IB4− neurons.

ASICs are highly Na^+^ selective channels over K^+^ and Ca_2_^+^, and eight ASIC subunits have been recognized (ASIC1a, ASIC1b, ASIC1b2 ASIC2a, ASIC2b, ASIC3, ASIC4 and BASIC) encoded by five different genes (ACCN1–5) in mammals (Hanukoglu, 2017; Vullo & Kellenberger, 2020). They are open when exposed to a broad range of pH level (from 4.5 to 7.0) depending on ASIC subunits (Hanukoglu, 2017; Osmakov et al., 2014). Among ASICs, ASIC1a, ASIC1b, ASIC2a, ASIC2b, and ASIC3 subunits are expressed in rat DRG neurons (Carattino & Montalbetti, 2020), and 90% ASIC2 is colocalized with ASIC3 in rat DRG neurons, generating functional heteromeric ASIC2‐3 channels, whereas homomeric ASIC2 and ASIC3 channels are expressed at a low level (Alvarez de la Rosa et al., 2002; Poirot et al., 2006; Waldmann et al., 1997). Considering that ASIC1a and ASIC3 largely existing in the sensory nerves play a functional role in regulating neural signaling conduction, in the present study respective antagonists to ASIC1a and ASIC3 were used to examine their effects for providing information of how ASICs are involved in the exercise pressor reflex (Liu et al., 2010). Our data showed the characterized ASIC1a‐like currents and ASIC3‐like currents recorded in rat muscle DRG neurons and that the respective inhibitors (PcTx1 to ASIC1a channels; APETx2 to ASIC3 channels) were confirmed to attenuate ASIC1a and ASIC3 currents. It is also noticed that, if double exponential function fitting τ_ieact_ of ASIC3‐like currents, some of ASIC3‐like currents showed one fraction with τ_inact_ of ~250 ms and another fraction with τ_inact_ over 2 s, which likely respectively corresponds to ASIC3 fraction and ASIC2 fraction as heteromeric ASIC2‐3 currents as described by Hattori et al. with ASIC2/3−/− KO mouse (Hattori et al., 2009). Further studies are necessary to be performed to identify heteromeric ASIC2‐3 channels in rat muscle DRG neurons.

Poirot et al. (2006) showed, in rat lumbar DRG neurons, ASIC currents are largely distributed in IB4− neurons as compared to IB4+ neurons, which is consistent with results in mouse lumbar DRG neurons observed by Dirajlal et al. (Dirajlal et al., 2003). Liu et al. showed that ASIC currents are evenly distributed in IB4− and IB4+ rat lumbar DRG neurons with different capsazepine sensitivity (Liu et al., 2004). A difference in results of those previous studies are likely due to that a selective group of DRG neurons responsive to capsacin were analyzed in Liu et al.'s experiments (Liu et al., 2004). In contrast, our current data showed that ASIC currents appear mostly in IB4− muscle DRG neurons, and that ASIC3‐like currents are more prominent than ASIC1a‐like currents in muscle DRG neurons (either IB4− neurons or IB4+ neurons). Nonetheless, our data indicate that functional ASIC1a and ASIC3 channels are expressed in rat muscle DRG neurons and the activities of their currents appear in IB4− muscle DRG muscle vs. in IB4+ muscle DRG neurons. It should be noted that our present study specifically examined the activities of ASICs in muscle DRG neurons, whereas DRG neurons in general as reported in the previous studies.

Of note, femoral artery ligation over 24 h increases ASIC3 protein expression and its current activity in rat muscle L4‐6 DRG neurons, but decreases ASIC1a protein expression (Farrag et al., 2017; Liu et al., 2010; Xing et al., 2012). It is agreed that upregulation of ASIC3 signaling pathways in muscle afferent nerves contributes to the exaggerated exercise pressor reflex in PAD. However, it is interesting that a blockade of ASIC1a can attenuate the BP response to muscle contraction in normal rats, but not in rats with femoral artery ligation (Ducrocq et al., 2020a, 2020b). Although accumulated evidence provided in the previous studies cannot be conclusive for the role of ASIC1a in regulating the exercise pressor reflex in PAD, data of our current study suggest that there is a discrepancy in ASIC1a and ASIC3 responsiveness in IB4− and IB4+ muscle DRG neurons, especially with a low pH, following femoral artery ligation.

Using the mice with brachial artery I/R, Ross et al. observed that I/R increases ASIC1a mRNA and ASIC3 mRNA in cervical and thoracic DRGs (C7/C8/T1) where the cell bodies of muscle afferents arising from the forelimb are located (Ross et al., 2014). This prior study further showed an exaggerated BP response in I/R mice during dynamic exercise (Queme et al., 2020). In decerebrated rats with femoral artery ischemia (6 h)/reperfusion (18 h), we also found an exaggerated exercise pressor reflex during static muscle contractions (Qin & Li, 2022). This result further suggested a necessary study determining ASIC currents in muscle DRG neurons under the I/R and FAO conditions. As indicated in the current study, the hindlimb I/R and FAO tended to increase the activities of ASIC1a‐like current in rat IB4− muscle DRG neurons and significantly increase ASIC3‐like currents. Additionally, we noticed that % distribution of ASIC1a currents is increased in IB4+ ASIC neurons of femoral artery I/R rat at pH 5.5, but not in IB4− ASIC neurons. Taken together, those results may be useful to clarify the role of ASIC1a and ASIC3 in regulating the exaggerated exercise pressor reflex in PAD rats with I/R injury and ischemia.

A previous study with ASIC subunit knockout mice has shown that channels with relatively fast kinetics (ASIC3‐like) are heterotrimers made of ASIC1, ASIC2, and ASIC3 (Gautam & Benson, 2013). Deletion of the ASIC3 subunit results in slow desensitization in channels. In our results, the effect of APETx2 (1 μM) in ASIC‐like currents was variable and it produced a modest inhibition. Similarly, PcTx1 (30 nM) partially inhibited ASIC‐like currents. Thus, ASIC currents observed in the current study was likely due to the activation of heteromeric ASICs after application pH 6.7 and pH 5.5 solutions.

Note that the exercise pressor reflex is equally important to be studied in females since both human and animal studies suggest that this reflexive response is lower in females than in males (Schmitt & Kaufman, 2003; Smith et al., 2019). A study design with simply female animals and determination of the role of female sex hormones in regulating ASIC currents can better clarify an issue on sex difference.

## 5 CONCLUSION

Overall, data of our current study suggest that limb I/R injury and ischemia in PAD can alter a distribution pattern of ASICs and also increase the activities of ASIC currents in muscle DRG neurons. Especially, limb I/R injury and ischemia amplify ASIC3‐like currents in a selective subpopulation of the muscle afferent neurons (expressing receptors for CGRP and depending NGF survival), which is likely a part of sensory signaling pathways involved in the exaggerated exercise pressor reflex in PAD.

## AUTHOR CONTRIBUTIONS

Q Li contributed to data collection and analysis of electrophysiology data and drafted the manuscript. L Qin participated in the analysis of data and drafting of the manuscript. J Li designed experiments, oversaw the performance of the experiments and data analysis, and drafted and revised the manuscript. All authors approved the final version of the manuscript submitted for publication.

## FUNDING INFORMATION

This study was supported by NIH R01 HL141198 and R01 HL164571 (to Jianhua Li), the American Heart Association Career Development Award (grant# 940567 to Lu Qin), and the Penn State College of Medicine Departmental DOM Innovation and Inspiration Award (INNOVQLI Fall2021 to Qin Li).

## CONFLICT OF INTEREST STATEMENT

The authors declare no conflict of interest.

## ETHICS STATEMENT

All experimental procedures were approved by the Institutional Animal Care and Use Committee of Penn State College of Medicine (Protocol#: PRAMS201147671) and were conducted in accordance with the National Institutes of Health Guide for the Care and Use of Laboratory Animals.
